# Intestinal Damage, Inflammation and Microbiota Alteration during COVID-19 Infection

**DOI:** 10.3390/biomedicines11041014

**Published:** 2023-03-27

**Authors:** Angela Saviano, Mattia Brigida, Carmine Petruzziello, Christian Zanza, Marcello Candelli, Maria Rita Morabito Loprete, Faiz Saleem, Veronica Ojetti

**Affiliations:** 1 Emergency Department, Fondazione Policlinico Universitario A. Gemelli, 00168 Roma, Italy; angela.saviano@policlinicogemelli.it (A.S.);; 2 Department of Gastroenterology, Policlinico Tor Vergata, 00133 Roma, Italy; 3 Emergency Department and Internal Medicine, San Carlo di Nancy Hospital, 00165 Roma, Italy; 4 Foundation “Ospedale Alba-Bra” and Department of Anesthesia, Critical Care and Emergency Medicine, Michele and Pietro Ferrero Hospital, 12060 Verduno, Italy; 5 Internal Medicine, Catholic University of the Sacred Heart, 00168 Roma, Italy

**Keywords:** ACE2, COVID-19, SARS-CoV-2, intestinal damages, microbiota

## Abstract

Background: The virus SARS-CoV-2 is responsible for respiratory disorders due to the fact that it mainly infects the respiratory tract using the Angiotensin-converting enzyme 2 (ACE2) receptors. ACE2 receptors are also highly expressed on intestinal cells, representing an important site of entry for the virus in the gut. Literature studies underlined that the virus infects and replicates in the gut epithelial cells, causing gastrointestinal symptoms such as diarrhea, abdominal pain, nausea/vomiting and anorexia. Moreover, the SARS-CoV-2 virus settles into the bloodstream, hyperactivating the platelets and cytokine storms and causing gut–blood barrier damage with an alteration of the gut microbiota, intestinal cell injury, intestinal vessel thrombosis leading to malabsorption, malnutrition, an increasing disease severity and mortality with short and long-period sequelae. Conclusion: This review summarizes the data on how SARS-CoV-2 effects on the gastrointestinal systems, including the mechanisms of inflammation, relationship with the gut microbiota, endoscopic patterns, and the role of fecal calprotectin, confirming the importance of the digestive system in clinical practice for the diagnosis and follow-up of SARS-CoV-2 infection.

## 1. Introduction

In the collective consciousness, COVID-19 infection is best identified by the respiratory symptoms associated with it; however, gastrointestinal symptoms have also consistently been shown to be an important component of COVID-19-related symptomatology. As early as 2020, a meta-analysis of 60 studies showed a collective prevalence of 17.6% for gastrointestinal symptoms in COVID-19 patients, and although various rates of incidence have been reported since then ranging up to 53%, such discrepancies may be attributable to regional variations [1]. Common gastro-intestinal symptoms include anorexia, abdominal pain, nausea, and vomiting. However, it may be observed that in the academic world, anorexia has not yet been universally accepted as a gastro-intestinal symptom in regards to infection by SARS-CoV-2 [2].

Research has thus far shown that the gastrointestinal tract is as much a target for the SARS-CoV-2 virus as the lungs. Indeed, gut enterocytes have been shown to be a major target of the virus and, through the help of both Angiotensin converting enzyme 2 receptors as well as transmembrane serine protease 2 (TMPRSS2) as a mediator, it is able to gain entry not only into the enterocytes of the ileum and colon, but also the gland cells of the esophagus [3]. In one study, gastro-intestinal symptoms preceded respiratory symptoms in 13% of cases [4]. In a large cohort of 20,133 in-patients surveyed across Britain, the rate of patients presenting exclusively with gastro-intestinal symptoms was found to be at 4%. Another smaller study with a cohort of 133 British patients had found a rate as high as 20% for patients presenting only gastro-intestinal symptoms.

The discovery that the virus is able to replicate within the cells of at least the rectum suggests that the SARS-CoV-2 virus infects the gastrointestinal tract [5,6]. It seems that the microbiological infiltration of the enterocytes and other cells of the gastrointestinal tract and the subsequent proliferation of the virus inside these cells is the most likely explanation for gastro-intestinal symptoms seen in COVID-19 patients [7]. Indeed, it also explains the presence of the SARS-CoV-2 viral load in fecal samples, the presence of which can also persist in fecal samples despite negative Oropharyngeal swab results [8]. In some cases, a SARS-CoV-2 viral load is detectable long after the virus can no longer be detected in the respiratory tract; in one pediatric case, gastro-intestinal samples continued to be positive up to 70 days after the respiratory viral shedding had ceased [9,10]. The presence of an RNA viral load in fecal matter consequently also explains the possibility of transmission of the virus through fecal matter [11,12].

## 2. Role of ACE2 Receptors in the Pathogenesis of COVID-19 Disease

**Figure 1:**
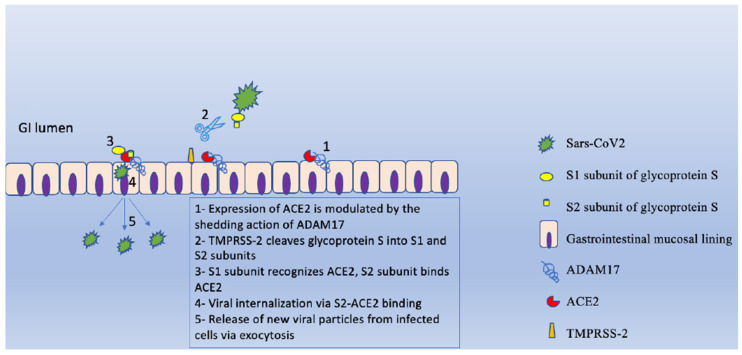
Illustration of the role of ADAM metallopeptidase domain 17 (ADAM17) and transmembrane protease, serine 2 (TMPRSS-2) in SARS-CoV-2 infection, adapted to the GI mucosal lining.

During SARS-CoV-2 infection, glycoprotein S (expressed on the virus membrane), after being processed by serine-protease TMPRSS2 (expressed on the host cell membrane), binds to ACE2 (Angiotensin Converting Enzyme 2). This protease cleaves glycoprotein S in two different subunits, S1 and S2, which are responsible, respectively, for the receptor recognition and for the fusion between this complex and the host cell’s membrane. In the S1 subunit, the C-terminal domain shows a high capacity to bind the ACE2; in this region, the SARS-CoV-2 receptor binding protein shows a binding capacity 10–20 times superior to previous SARS-CoV-2 [13]. Furthermore, the action of two proteases, namely ADAM17 (ADAM metallopeptidase domain 17) and TMPRSS-2 (transmembrane protease, serine 2), is necessary in order to allow the viral and host membrane fusion through the S2 subunit [14,15,16]. Next, the internalization of SARS-CoV-2 via endocytosis occurs, followed by viral RNA release for replication and translation by the host cell machinery, which further assembles and releases new viral particles via exocytosis (Figure 1).

Moreover, SARS-CoV-2 RBD binds to soluble hACE2 strongly [17]. The affinity for hACE2 may be responsible for SARS-CoV-2’s higher infectivity [18]. The ACE2 receptors are highly expressed both on the lung alveolar and small intestinal epithelial cells, but also on vascular endothelial and smooth muscle cells, in kidneys, skin and in the oral and nasal mucosa [19]. ACE2 cleaves Ang II to Angiotensin (1–7), which has vasodilating, anti-inflammatory and anti-fibrotic effects through the binding with Mas receptors [20]. This enzyme functionally counteracts the physiological role of ACE, the homolog enzyme that cleaves Angiotensin I into Angiotensin II. It plays a key role in vasoconstriction, renal sodium reabsorption and potassium excretion, the synthesis of aldosterone, the elevation of blood pressure and the induction of pro-fibrotic and inflammatory processes. Evidence shows that the availability of different Angiotensin peptides and therefore the balance between anti-inflammatory and pro-inflammatory pathways is determined by the ACE/ACE2 balances [19]. SARS-CoV-2 determines a disruption of this ACE/ACE2 balance and activation of RAAS, which ultimately leads to the progression of COVID-19, particularly in comorbid patients (cardiovascular disease, hypertension, diabetes mellitus) [21]. In patients with Inflammatory Bowel Disease (IBD), a different expression of ACE2 receptors on intestinal mucosa has been demonstrated [22]. Patients with active IBD expressed higher ACE2 protein (detected with immunohistochemical analyses) in terminal ileum and colon compared with controls. Moreover, the average expression of soluble ACE2 resulted increased in patients with IBD (mainly in Crohn Disease) [23]. As mucosal inflammation increases the ACE2 presence, it could be assumed that IBD patients have more severe COVID-19. However, in a study from Wuhan, which studied 318 patients with IBD during a local outbreak, no COVID-19 cases were detected [24], although the results might be also related to the local adjustment of protection to prevent the infection [24]. A recent study analyzes the expression of ACE receptors in the small bowel epithelium in subjects affected by Crohn’s Disease, ulcerative colitis (UC) and controls. It resulted that a lower expression of ACE2 (as detected in UC patients) was associated with poor COVID-19 outcomes, while an elevated expression of ACE2 was associated with worse outcomes. It has been proposed that ACE2 may play a paradoxical role in the disease progression of COVID-19. They both increase viral uptake and showed anti-inflammatory properties [25]. The study also suggests that the restoration of ACE2 expression by the use of an anti-cytokine therapy might be important in the infection of SARS-CoV-2 and might relate to a lower morbidity of COVID-19 [25]. Recent studies also suggested that the soluble form of ACE2 acts as a competitive binding, preventing the binding of the SARS-CoV-2 virus to the full-length ACE2 protein [26]. Interestingly, the cleavage of ACE2 into the soluble form is regulated by the TNF-alpha convertase ADAM17, a protease upregulated in active IBD [27]. As binding between glycoprotein S and ACE2 is such a crucial step in COVID-19 pathogenesis, several studies analyzed the connection between the use of ACE inhibitor drugs and SARS-CoV-2 infection. ACE inhibitors and Angiotensin receptor blocker (ARB) drugs are widely used to treat congestive cardiac failure and hypertension in adult patients [28]. The association between these drugs and COVID-19 was, at first, uncertain, which led to argument of whether patients taking these drugs had to change their therapy when being infected by SARS-CoV-2. A prospective, multicentric study, led by the group of Hakeam, enrolled a group of 338 patients who had been recovering in hospital because of COVID-19, all of them under ACE inhibitor prescriptions, with only 197 continuing therapies during hospitalization. Results show that patients with cardiovascular disease using ACE inhibitors and ARB therapies are not at a higher risk of severe COVID-19 on their admission at the hospital, and maintaining the therapy during hospitalization is associated with a lower risk of death [28]. A cohort study including 8.3 million people, led by the Cox and Watkinson group, attempts to find out if patients prescribed ACE inhibitors or ARBs had a higher risk of severe SARS-CoV-2 infection with intensive care unit (ICU) admission. The authors concluded that ACE inhibitors and ARBs are associated with a reduced risk of COVID-19 disease without a significantly increased risk of ICU admission [29].

## 3. Inflammation, Acute and Chronic GI Conditions and the Role of Endoscopy in COVID-19 Patients

Patients who suffer from a chronic gastrointestinal condition may be at an increased risk of developing a more severe form of COVID-19. This may be related to the patients’ coexisting comorbid conditions, the chronic pharmacologic regimen associated with the particular GI chronic disease and the pre-existing inflammatory state per se in the GI tract [30].

For example, a very wide cross-sectional survey on more than 85,000 patients gives us an idea of the correlation between chronic pharmacological history and COVID-19. In this study, where more than 50% of patients reported acid reflux and heartburn and a chronic use of PPI, reveals a significant dose-dependent correlation between PPI use and the odds of reporting a positive COVID-19 test result [31]. This raises an open question on how a luminal pH increase associates with a risk modification of COVID-19 colonization in a mainly respiratory system-targeting virus, and further studies would be needed to better validate this mentioned result.

Moreover, even though studies on IBD patients have not reported a higher prevalence of COVID-19 compared to the general population, IBD-related use of glucocorticoids (but not anti-TNF-alpha treatment) may be associated with a higher risk of a more severe COVID-19 infection in this subgroup of gastroenterological patients [32,33,34]. Indeed, an expert commentary from the American Gastroenterological Association (AGA) published at the beginning of the COVID-19 pandemic recommended using glucocorticoids for a short course and at the lowest dose needed for achieving a clinical response, before prescribing a glucocorticoid-sparing treatment [35].

In contrast, concerning acute GI symptoms in patients with COVID-19, different etiological mechanisms have been described. Direct damage can be caused at the cellular level, with the invasion of ACE-2-expressing cells by SARS-CoV-2, and, along with this, a form of indirect damage can also be caused by the resulting inflammatory response [9]. The second of the two mechanisms of inflammation may be described as acting through immune system activation and, at times, cytokine storm syndrome (CSS) [36].

A state of hyper-inflammation and cytokine storm syndrome in patients can exacerbate their condition and complicate the ongoing therapeutic approaches [37]. The process involves the infected cells releasing a large number of inflammatory markers and cytokines such as Interleukin 2 and 7, tumor necrosis factor-alpha, granulocyte colony stimulating factor, interferon gamma inducible protein 10, monocyte chemoattractant protein 1 and macrophage inflammatory protein 1 alpha [38,39]. The overproduction of these markers is also known as a “cytokine storm” and it is this storm that causes an accumulation of immune cells in the gastro-intestinal tract. To that extent, a large number of lymphocytes, plasma cells and interstitial edemas have been found in the layers of the gastro-intestinal tract [40]. The severity of the disease and any ensuing multiple organ insufficiency correlates with this abnormal release of cytokines [41]. It is in the suppression of this disproportionate immune response that immunosuppressors have been found to be a useful addition to COVID-19 therapeutic approaches.

A single-center observational study on around 180 patients with acute respiratory distress syndrome (ARDS) found higher rates of ileus and bowel ischemia in ARDS-COVID-19 positive patients compared to ARDS-COVID-19 negative individuals [42]. These two subgroups were matched for age, comorbidities and SOFA score (Sequential Organ Failure Assessment score) on intensive care unit admission, and even though they were not matched by inflammatory markers, a cytokine storm underlying COVID-19 once again could be considered as a factor influencing prognosis in these patients.

**Figure 2:**
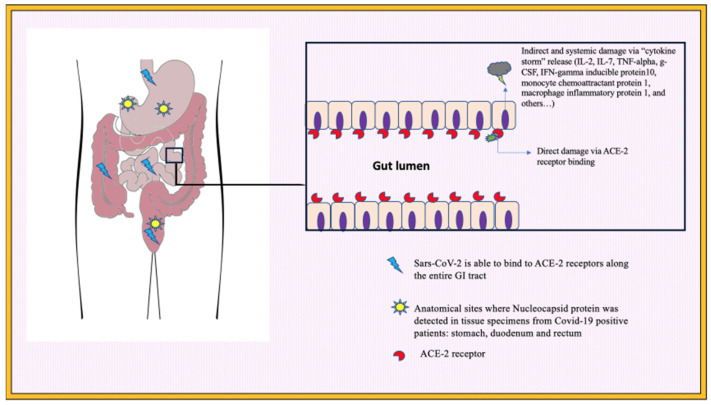
Simplified representation of mechanisms of SARS-CoV-2-related damage and histopathology findings in COVID-19 patients with GI symptoms.

The two forms of damage described (i.e., direct and indirect) have been identified as some of the underlying causative factors in the pathogenesis of diarrhea in COVID-19 (Figure 2), although the exact mechanism is still unclear. Diarrhea may be related to the effects of the damage on compromised cells and their resulting change in intestinal secretions, intestinal permeability and a subsequent change in the absorption of fluids [9].

Moreover, intestinal hypoxia has also been shown to increase the expression of ACE2 and TRFC genes and hence may drive intestinal inflammation and worsen prognosis [43].

Limited evidence in the literature has described a more favorable outcome and viral RNA detection in stools in patients with COVID-19 and concomitant diarrhea [11,44,45]. For instance, a cohort study conducted on almost 200 hospitalized patients with COVID-19, in-hospital mortality reported in those with diarrheal symptoms was lower compared to patients without diarrhea [46]. This has led authors to suppose that the virus preferentially targeted the mucosa of the GI tract rather than the respiratory system, in patients with a milder COVID-19 course and concomitant GI symptoms.

This evidence further motivates the scientific interest in endoscopically identifying what types of mucosal damages are present in this subgroup of COVID-19 patients.

However, the role of endoscopy in COVID-19 patients with gastrointestinal manifestations is not completely clear. Endoscopy is an important diagnostic tool in patients presenting with more severe gastro-intestinal symptoms useful to discover a major finding within seven days of onset of COVID-19 [47].

Gastroscopies are useful in selected patients with gastro-intestinal bleeding and findings most often can include esophageal, gastric and duodenal ulcers along with erosions, different patterns of inflammation, oedema and ecchymoses [47,48,49,50,51]. Moreover, SARS-CoV-2 RNA has been found in biopsies of the esophagus, stomach, duodenum and rectum. In particular, histopathology reports from tissue specimens in COVID-19 patients revealed a main distribution of viral detection in the cilia of epithelial glandular cells, intestinal and gastric epithelial cells, with the viral nucleocapsid protein being detected in glandular epithelial cells from the stomach, duodenum and rectum (Figure 2) [52]. Literature studies reported that most gastroscopy results were normal, different to colonoscopies (only a small number of colonoscopies were found to be normal) [47]. Colonoscopies in COVID-19 patients showed non-specific inflammation, ulcerative inflammatory colitis, ischemic colonopathy and non-specific erythema [47,51].

Nonetheless, the overall number of endoscopic examinations decreased during the pandemic period. Indeed, a safety approach internationally adopted for the endoscopic outpatient setting consisted of testing patients for COVID-19 before they could undergo endoscopy [53,54,55], thus postponing an endoscopy for those patients resulting positive for this viral infection. This implies that the endoscopic evidence mainly available in the literature comes from an endoscopy on hospitalized patients, thus drastically reducing the possibility of characterizing mucosal lesions on a larger scale in the overall category of patients.

Kuftinec et al. [56] reported that of a total of 1992 patients, only a small percentage 1.2% (24 patients) underwent endoscopic procedures. All of these patients were also admitted to the intensive care unit. Of them, only 10 required an intervention for either bleeding, and/or enteral access for a biliary obstruction. The majority of endoscopic findings were considered to be related to a chronic illness more than to direct viral gastrointestinal damage.

Emara et al. [57] focused on the impact of COVID-19 on endoscopic exams, revealing a significant reduction of procedures during the pandemic period. Vanella et al. [47] registered endoscopic abnormalities and risk factors in COVID-19 patients. These authors conducted a multicenter (16 institutions) cross-sectional study between February and May 2020, reporting that 106 COVID-19 patients performed endoscopies; 33% of them were admitted in intensive care units; 44.4% of them have reported gastrointestinal symptoms. Of all endoscopies, 66.7% were urgent (mainly for gastrointestinal bleeding) and 45.6% of patients revealed major abnormalities: in particular, ulcers (25.3%), erosive gastro-duodenopathy (16.1%) and hemorrhagic gastropathy (9.2%). Among lower GI endoscopies, 33.3% showed an ischemic-like colitis. Interestingly, elevated D-dimers >1850 ng/mL resulted to better predict major mucosal injuries and abnormalities in COVID-19 patients. Finally, Pohl [58] in his paper suggested that the COVID-19 pandemic taught that a high proportion of procedures often are performed unnecessarily, and it is important to reassess the current practice in order to better tailor the indication of these procedures to patients who really can benefit from them.

## 4. Calprotectin

As an acute-phase protein, calprotectin has been used as a valuable tool in treating and diagnosing diseases such as inflammatory bowel disease through fecal, serum and plasma samples. Previous research has helped to extend its use by showing the relationship of calprotectin and SARS-CoV-2 in COVID-19 patients with pneumonia. Showing that plasma and fecal calprotectin could potentially be used as a marker of inflammation in COVID-19 [59]. A more recent meta-analysis conducted on eight cohort studies with a pool of 805 patients showed that plasma calprotectin could also be assumed as a marker of severity in COVID-19 patients, as it consistently showed significantly higher values (greater than 24.1 mg/L) in patients more severely affected by disease, confirming similar findings in an older meta-analysis study [60]. Another study with a cohort of 89 individuals, although confirming the valuable use of calprotectin as a prognostic indicator, showed that, fundamentally, calprotectin levels were not reflective of the severity of gastrointestinal symptomatology. The COVID-19 patients who had presented in the study with diarrhea did not show increased levels of fecal calprotectin [61].

A recent interventional study performed by Ojetti et al. [59] tests the efficacy of a mix of probiotics in hospitalized patients with COVID-19 pneumonia. This randomized study confirms the correlation between COVID-19 interstitial pneumonia and high levels of fecal calprotectin (expression of a worse prognosis), regardless of the presence of gastrointestinal manifestations, as demonstrated previously. Moreover, in the group supplemented with the probiotics, they observed a more rapid decrease in inflammatory markers such as fecal calprotectin and C reactive protein and a slight, not significant, decrease in the oxygen support.

## 5. Role of the Microbiota and the Immune System

Although the causality is still unclear and may remain so until future longitudinal studies, some studies have found that gut dysbiosis in COVID-19 patients and an increase in the number of opportunistic pathogens [62,63,64,65]. The involvement of cytokine responses in SARS-CoV-2 infections may also be an element that drives changes in gut microbiota [66]. In addition, the level of severity of disease has been found to correlate with gut dysbiosis [62]. However, the level of effect that secondary infections may play cannot be ruled out [63]. Furthermore, COVID-19 patients may be more susceptible to dysbiosis due to diarrhea; a change in intestinal flora may result in severe illness as inflammatory factors are linked to the gut microbiome [64,65,66]. The link between the gut microbiome and lungs has been established in emerging studies and is often referred to as the gut–lung axis [67,68,69]. Whatever the cause of dysbiosis, the result can nonetheless disturb the gut–lung axis. There is evidence to suggest that a compromised gut–lung axis can increase the susceptibility to respiratory disease and cause modifications in the immune responses and homeostasis of the lungs [70,71,72,73]. The coexpression of ACE-2 in alveolar type 2 cells and intestinal and colonic epithelial cells, the consequent viral replication not limited to the respiratory system but also involving the gut and the clinical presentation of SARS-CoV-2-infected patients often including diarrhea other than the classical respiratory symptoms, are all elements pointing towards a possible link between the microbiota and the current pandemic infectious disease known as COVID-19.

In this regard, estimates report the presence of viral RNA in up to 48% of fecal samples or rectal swabs from COVID-19 patients [40,74,75].

In wellbeing conditions, the gut and the lung microbiota share some of the microbial phyla, such as *Proteobacteria*, *Actinobacteria*, *Bacteroidetes* and *Firmicutes* [76,77]. Despite an overall difference in terms of compositional and functional microflora in these two organs, both their microbiota systems play a role in mediating the systemic and local inflammatory responses, thus contributing to host homeostasis via the so-called “gut–lung axis”. In this scenario, bronchial-associated lymphoid tissue and gut-associated lymphoid tissue, also known as mucosal-related immune systems, are the actors of the gut–lung axis’s immunomodulatory effects [70,78,79].

An interesting study by Prasad et al. on the plasma microbiome in COVID-19 subjects showed high serum levels of lipopolysaccharide, peptidoglycan and fatty acid-binding protein 2 (markers of gut permeability) in these patients compared to controls, highlighting a precarious intestinal barrier state during the infection [80].

It has also been hypothesized that SARS-CoV-2 can migrate from the lungs to other tissues, including the gastrointestinal tract, using cells of the immune system as a transport route as it has been reported for influenza viruses [81,82,83]. Once the virus reaches the epithelial cells of the intestine, it will bind to them via ACE-2, causing the release of chemokines and cytokines. Subsequently, the development of an inflammatory cascade in the intestine is characterized by the infiltration of macrophages, neutrophils and T cells, which determines a further dysbiosis [84,85,86]. In more detail, these innate immune cells will bind to pathogen-associated molecular patterns via the pattern recognition receptors, and signaling pathways such as NF-kB, JAK/STAT and IRF3 promote the expression of interferons (INF), IFN-stimulated genes and pro-inflammatory factors, and this will finally lead to an excessive release of inflammatory cytokines. Furthermore, a condition of dysbiosis is instead harmful on the thigh junctions of the gut barrier, and the production of inflammatory mediators such as TNF-alpha, IL-2, IL-6, IL-10, IL-18 and CXCL10 is favored in the context of this impaired microbiota balance. On the other hand, the gut microbiota eases inflammasome activation, dendritic cell migration, the expression of pro-IL 1 beta and pro-IL-18, thus supporting a protective immunity after viral infection. Moreover, the intestinal microbiota exerts antiviral effects within the lung via IFN-alpha and IFN-beta, by regulating the IFN-I receptor expression in the respiratory epithelium [81].

Different data coming from Asian studies have described the characterization and possible implications of the microbiota composition of subjects affected by COVID-19. For instance, Yeoh et al. [87] conducted a bicentric cohort study where they collected patient records and sampled blood and stool from 100 individuals infected with SARS-CoV-2, in order to characterize the microbiota composition via shotgun sequencing of nucleic acids extracted from stools, and to measure the levels of inflammatory cytokines and blood markers. For some of these patients, the authors collected stool samples up to one month after the clearance of infection. Their results show an underrepresentation of *Bifidobacteria*, *Eubacterium rectale* and *Fecalibacterium prausnitzii* and these concentrations were low in those samples collected at one month from the SARS-CoV-2 infection resolution. Furthermore, the degree of dysbiosis seemed to correlate with the disease severity consistently, with high levels of C-reactive protein, aspartate amino-transferase, gamma-glutamyl transferase, lactate dehydrogenase and inflammatory cytokines such as TNF-alpha, IL-10, CCL2 and CXCL10. Another significant message from these authors is that the gut microbiota of COVID-19 patients seems to be altered either in those treated and in individuals not treated with antibiotics [87]. However, it is still debated whether inflammatory-associated dysbiosis in COVID-19 patients has an active role in the disease severity or simply represents an opportunistic event.

These findings were confirmed in a smaller cohort by Zuo et al. [63], who conducted fecal metagenomic sequencing from samples of 15 patients hospitalized for COVID-19.

These authors not only found that dysbiosis was correlated with the disease severity (particularly, an abundance of *Clostridium hathewayi*, *Clostridium ramosum* and *Coprobacillus* and decreased levels of *Faecalibacterium prausnitzii*) but they also described the persistence of an altered microbiota composition even after the resolution of infection and related symptoms. A peculiar finding from this study [63], once again linking the gut to COVID-19, is that the levels of *Bacteroides ovatus*, *Bacteroides dorei*, *Bacteroides massiliensis* and *Bacteroides thetaiotaomicron* during hospitalization (the same species that showed to downregulate intestinal expression of ACE-2 in murine gut) were inversely correlated to the load of SARS-CoV-2 in the stool samples from these patients, once again suggesting an implication of the gut microbiota in COVID-19 pathogenesis [63].

## 6. Probiotics’ Role in Patients with COVID-19 Infection

Intestinal permeability and the intestinal microbiota represent an important element both in modulating the absorption of nutrients and in acting as a barrier against the passage of noxious substances from the lumen into the bloodstream. It has been demonstrated that probiotics can regulate the composition of the microbiota and therefore contribute to the maintenance of homeostasis. Although the role of probiotics during the pandemic has not been sufficiently codified within clinical practice, this aspect has nevertheless been explored within the scientific community, together with the possible role of gut–microbiota modulation in COVID-19 [88,89,90]. Gutierrez and colleagues produced data on probiotic use in COVID-19 patients, demonstrating that they could improve not only gastrointestinal symptoms such as diarrhea, but also cough and headache, in support of the gut–lung axis microbiota association [90,91,92]. Moreover, these authors demonstrated that probiotics were associated with a significant increase in complete viral clearance and remission of symptoms within 30 days. Furthermore, significant effects were also observed in reducing the duration of symptoms and lung infiltrates with better outcomes. No significant changes were noticed in the fecal microbiota composition after the use of probiotics. The authors hypothesized that the probiotic strains used could primarily act on the gut–lung axis, interacting with the immune system and stimulating humoral immunity with beneficial effects. A study of Ojetti et al. [93] found that the supplementation with a mix of probiotics (*Bifidobacterium lactis LA 304*, *Lactobacillus salivarius LA 302* and *Lactobacillus acidophilus LA 201*) for 10 days in patients with COVID-19 interstitial pneumonia significantly reduced gut inflammatory markers and abdominal symptoms, too.

Sandrayee Brahma et al. [94], in their study, highlighted that the gut dysbiosis due to COVID-19 infection may potentially have “long-term” gut and lung health implications, favoring an increase in opportunistic pathogens and an immune dysregulation that can remain up to six months post infection. So, supplementation with probiotics including *Lactobacillus* and *Bifidobacterium* could act as a positive immune modulator, also restoring the gut dysbiosis and contributing to giving benefits for both respiratory infection and gastrointestinal symptoms. Nguyen et al. [95] concluded that the use of probiotics (in particular, they studied *Lactobacillus plantarum* and its metabolites) could play a complementary strategy besides vaccines in fighting COVID-19 infection for some antiviral effects. Finally, Khaled et al. [96] speculated about the possible biological role of probiotics against the dangerous cytokine storm related to COVID-19 infection. Therefore, more research and clinical and laboratory investigations are required to explore this field and many questions remain unanswered.

## 7. Conclusions

This review summarizes data on SARS-CoV-2’s effects on gastrointestinal systems, including the mechanisms of inflammation, the relationship with gut microbiota, endoscopic patterns, the role of fecal calprotectin confirming the importance of the digestive system in clinical practice for the diagnosis and follow-up of SARS-CoV-2 infection.

The knowledge of physio-pathological mechanisms through which SARS-CoV-2 infects the lungs and intestinal cells is essential to discover and develop new therapeutic targets and to guide research in the early understanding of the severity and evolution of infection, predicting the outcomes of our patients.

## Data Availability

No new data were created or analyzed in this study. Data sharing is not applicable to this article.
